# Contextual analysis of hematopoietic stem cell transplantation in outpatients

**DOI:** 10.1590/0034-7167-2025-0173

**Published:** 2026-08-21

**Authors:** Camila Caroline Marcolino Soares, Valéria Dantas de Azevedo, Luana Silva Pereira Sátiro, Edmundo João Mansilla-Cordeiro, Viviane Euzébia Pereira Santos, Isabelle Campos de Azevedo

**Affiliations:** I Universidade Federal do Rio Grande do Norte. Natal, Rio Grande do Norte, Brazil

**Keywords:** Outpatients, Ambulatory Care, Transplantation, Hematopoietic Stem Cell Transplantation, Bone Marrow Transplantation., Pacientes Ambulatorios, Atención Ambulatoria, Trasplante, Trasplante de Células Madre Hematopoyéticas, Trasplante de Médula Ósea.

## Abstract

**Objectives:**

to analyze and map the contextual aspects of hematopoietic stem cell transplantation in outpatients in the literature.

**Methods:**

a scoping review was conducted from October 2023 to June 2024 in databases, portals, and thesis and dissertation libraries. Data collection and analysis were based on the framework proposed by Hinds, Chaves, and Cypress, according to the contextual layers: metacontext, general, specific, and immediate.

**Results:**

the final sample consisted of 30 studies. Regarding the contextual layers, metacontext addressed outpatient hematopoietic stem cell transplantation worldwide; general, advantages and weaknesses; specific, effects of these care models; and immediate, characteristics of patients undergoing outpatient transplantation.

**Final Considerations:**

it was observed that outpatient transplantation is a healthcare trend that offers benefits for improving patients’ health, but faces challenges in its implementation.

## RESUMEN

**Objetivos:**

analizar y mapear los aspectos contextuales del trasplante de células madre hematopoyéticas en pacientes ambulatorios en la literatura.

**Métodos:**

se realizó una revisión exploratoria entre octubre de 2023 y junio de 2024 en bases de datos, portales y bibliotecas de tesis y disertaciones. La recopilación y el análisis de datos se basaron en el marco de Hinds, Chaves y Cypress, según los niveles contextuales: metacontexto, general, específico e inmediato.

**Resultados:**

la muestra final constó de 30 estudios. En cuanto a los niveles contextuales, el metacontexto abordó el trasplante ambulatorio de células madre hematopoyéticas a nivel mundial; a general, las ventajas y debilidades; a específico, los efectos de estos modelos de atención; y a inmediato, la caracterización de los pacientes sometidos a trasplante ambulatorio.

**Consideraciones Finales:**

se observó que el trasplante ambulatorio es una tendencia de atención médica que ofrece beneficios para mejorar la salud de los pacientes, pero enfrenta desafíos en su implementación.

## INTRODUCTION

Hematopoietic stem cell transplantation (HSCT) is a therapy indicated for various types of oncohematological diseases, consisting of the infusion of hematopoietic stem cells (HSCs) to reestablish bone marrow function so that it produces new healthy cells^(1)^.

Traditionally, this treatment was usually performed entirely in a hospital setting, as there was the infrastructure and equipment necessary to care for these patients^(2)^. However, with advances in scientific research in the field, the development of outpatient HSCT has been considered a new, safe, and viable therapeutic option compared to transplants performed entirely during hospitalization^(2,3)^.

In outpatient HSCT, patients do not need to be hospitalized, returning daily to the clinic where they receive care in all phases of treatment, namely pre-HSCT, Day Zero, and post-HSCT^(4,5)^. Thus, patients experience a daily routine of chemotherapy, transfusions, central venous catheter care, and multidisciplinary assessment until bone marrow transplantation and discharge^(5)^.

This model requires that the patient and caregiver be trained to identify warning signs, such as fever, bleeding, or changes in general condition, in order to allow for early and safe interventions, ensuring continuity of treatment until engraftment, which occurs between ten and 30 days after the infusion of HSCs. After confirmation of engraftment and clinical stabilization, patients undergo periodic outpatient follow-up to control long-term complications and ensure transplant success^(6)^.

It is also important to highlight that, for the success of outpatient HSCT, a joint, harmonious, and cohesive action is necessary from a multidisciplinary team that works daily providing guidance on nutrition, hygiene, and medication administration at home, in addition to offering psychological and pharmaceutical support, reinforcing the importance of adherence to treatment and protective home isolation^(3,6)^.

As members of this team, nurses are present throughout the patients’ entire treatment. Therefore, these professionals must possess scientific knowledge that enables them to apply care methods appropriately, demonstrating mastery over transplant patients’ actions and needs, which will contribute to their recovery^(6)^.

Nurses have the responsibility to intensify care, maintain constant attention to risks and other complications, adopt strategies that promote patient self-care, guide family members regarding the needs involved in this process, and ensure effective communication with the interdisciplinary and multidisciplinary team^(7)^.

However, even with the benefits for healthcare institutions and patients, this care model is not yet a reality practiced by healthcare services in Brazil and worldwide, as it has a low adoption rate, such as in the United States of America (USA), which has 45% of outpatient HSCT clinics, and the United Kingdom, where only 19% of oncohematological treatment units are outpatient clinics^(8)^.

Given the above, this study is justified because outpatient HSCT is a relatively new healthcare practice that needs to be better elucidated: in what contexts does it occur, what are the reasons that favor its development, and what are the challenges for its implementation? These factors are crucial for the overall interpretation of the phenomenon investigated so that it can be replicated by other countries.

To this end, context analysis is a theoretical and methodological framework that favors this investigation, as it helps healthcare professionals understand human beings, assisting in the development of conditions for health promotion and meaningful experiences. Through a purposeful, systematic, and analytical relationship with the phenomenon, the aim is to understand its meaning in its totality and to conduct a global analysis. Therefore, elucidating a context will enable the prediction, explanation, and comprehension of the investigated phenomenon^(9)^.

Thus, context can be defined as a set of interconnected relationships related to an event or scenario. To analyze it, it is necessary to understand this phenomenon at four interrelated interactive levels: metacontext, general context, specific context, and immediate context^(9)^.

From this perspective, it is important to conduct research that identifies and analyzes the contextual aspects of this type of outpatient treatment in order to present its main characteristics, benefits, challenges, and to elucidate how it is carried out worldwide.

Therefore, the following guiding question was defined: what are the contextual aspects of HSCT in outpatients?

## OBJECTIVES

To analyze and map the contextual aspects of HSCT in outpatient settings within the literature.

## METHODS

### Ethical aspects

Approval from the Research Ethics Committee was waived, as this was a review of data available in the literature and did not involve human subjects. However, it should be noted that all citations of the mapped studies were duly carried out and respected.

### Study design

A scoping review was developed, in accordance with Preferred Reporting Items for Systematic Reviews and Meta-Analyses extension for Scoping Reviews^(10)^ guidelines and JBI recommendations^(11)^.

This type of review aims to identify, map, and synthesize the evidence and gaps in knowledge surrounding a particular phenomenon. The process follows a rigorous methodological approach outlined in the following stages: defining the objective and research question; aligning inclusion and exclusion criteria with the study objective and research question; describing the search for evidence, selection, data extraction, and presentation of findings; searching for evidence; and selection, extraction, analysis, synthesis, and presentation of evidence^(11)^.

It is worth noting that the method proposed by JBI^(11)^ was applied to develop the study protocol, define the objective, research question, eligibility criteria, search different data sources and map evidence aligned with the object of study in an organized and systematic way. The protocol was developed and duly registered in the Open Science Framework (OSF) (DOI: https://doi.org/10.17605/OSF.IO/8GSZN).

### Study period and location

The search in databases and grey literature was conducted between October 2023 and updated in June 2024. Data collection took place in databases (U.S. National Library of Medicine (PubMed), Scopus, Web of Science, Science Direct, Latin American and Caribbean Literature in Health Sciences (LILACS), Cochrane), in thesis and dissertation catalogs (Academic Archive Online (DIVA), Education Resources Information Center (ERIC), DART-Europe E-Theses Portal, Electronic Theses Online Service, National ETD Portal, *Teses e Dissertações da América Latina*, Coordination for the Improvement of Higher Education Personnel (In Portuguese, *Coordenação de Aperfeiçoamento de Pessoal de Nível Superior* - CAPES) Thesis and Dissertation Portal), and in a digital library (National Library of Australia’s (Trove)).

### Inclusion and exclusion criteria

Eligibility criteria were defined based on the expertise and experience of the members and researchers of the research group, who, through consensus, decided to include scientific articles indexed in health journals, theses and dissertations, since evidence is produced from robust scientific methods, well-defined with theoretical depth and that have a substantial innovative contribution to the scientific field under study.

These studies should address the study’s objective and guiding question, be available in full electronically, without temporal or language restrictions, accessible through the Federated Academic Community via the CAPES Journals Portal of the Ministry of Education. Studies in the form of editorials, letters to the editor, books, book chapters, abstracts, and opinion articles were excluded. Duplicate documents were considered only once.

### Study protocol

Before initiating the protocol development process, an open literature search and an open search of the OSF were conducted to verify the existence of previously published studies or any similar study protocols under development, which were not found.

Formulating research questions to investigate or assess a clinical problem aims to understand the complexity of care and disseminate this knowledge among the scientific community and patients^(9)^. Therefore, to formulate the guiding question, the PCC strategy was used (Population: patients; Concept: HSCT; Context: outpatient clinic). Thus, the guiding question determined for the search for evidence was: what are the contextual aspects of HSCT in outpatients?

Subsequently, a search for studies was conducted in PubMed and Cochrane to identify the descriptors and keywords that best suited the object of study. Descriptors were selected in English using Medical Subject Headings, and in Portuguese using the Health Sciences Descriptors. Additionally, the Boolean operators AND and OR were used. Thus, the following search strategy was defined in English, as presented in Chart 1, which was adapted according to the specificities of each data source.

**Chart 1:** Search strategy, 2025

	P - Participants	C - Concept	C - Context
**Extraction**	Outpatient	Transplantation	Hematopoietic stem cell transplantation
**Conversion**	Outpatient	Transplantation; grafting	Hematopoietic stem cell transplantation; Bone marrow transplantation; stem cell transplantation
**Construction**	Outpatient	transplantation OR grafting	“hematopoietic stem cell transplantation” OR “bone marrow transplantation” OR “stem cell transplantation”
**Use in databases and catalogs of theses and dissertations**			
**U.S. National Library of Medicine**	((outpatients[Title/Abstract]) AND (transplantation[Title/Abstract] OR (grafting[Title/Abstract]))) AND (“hematopoietic stem cell transplantation”[Title/Abstract] OR (“bone marrow transplantation”[Title/Abstract] OR “stem cell transplantation”[Title/Abstract]))		
**Cochrane**	outpatients in Title Abstract Keyword AND transplantation OR (grafting) in Title Abstract Keyword AND “hematopoietic stem cell transplantation” OR (“bone marrow transplantation” OR “stem cell transplantation”) in Title Abstract Keyword		
**Coordination for the Improvement of Higher Education Personnel**	outpatients AND transplantation OR (grafting) AND “hematopoietic stem cell transplantation” OR (“bone marrow transplantation” OR “stem cell transplantation”)		

The searches were initiated, and the results were exported to Rayyan^®^ software^(12)^, which assisted in the pre-analysis stage of the titles and abstracts. Subsequently, the selected studies were read in full and subjected to the eligibility criteria listed. The stages were carried out by pairs of reviewers with expertise in HSCT, and in cases of disagreement, the opinion of a third reviewer was requested.

### Data analysis

After selecting the studies, the reviewers used a protocol developed to extract the following information: data sources; title; authors; year of publication; country where the study was conducted; methodological design; objective; type of HSCT; and contextual layers (metacontext, general context, specific context, and immediate context). The collected data were organized in a spreadsheet using Microsoft Excel^®^ 2017 software.

To identify the contexts and analyze the evidence, the theoretical-methodological framework of contextual analysis proposed by Hinds, Chaves and Cypress^(9)^ was followed, which allows examining the relationships that constitute a phenomenon to make its occurrence and extent more understandable in four contextual layers: metacontext, general context, specific context, and immediate context^(9)^.

Metacontext incorporates socially constructed knowledge, highlighting the present and emphasizing the conditions and lessons learned as a model for the future, with direct explanation and influence on the behaviors and events of that particular phenomenon^(9)^. Therefore, at this level, information was sought regarding existing guidelines, policies, or other documents concerning the implementation of outpatient HSCT.

General perspective encompasses interpretations conceived through past and present interactions of the phenomenon over time^(9)^. Therefore, the aim was to highlight the advantages and weaknesses of these models for healthcare services. Specific encompasses the immediate past and the circumstances that influence the present moment, such as time, people, location, among others^(9)^. Thus, the aim was to map the positive effects of these outpatient HSCT models.

Immediate context focuses on the present and expresses the phenomenon in question, and can be defined by relevant aspects of the phenomenon, such as space, action, and protagonists, which will delimit the pattern of behavior^(9)^. In this layer, the main characteristics related to outpatient HSCT were identified.

Evidence was grouped into the corresponding contextual layers in accordance with the method proposed by Hinds, Chaves and Cypress^(9)^, and presented by means of an image that illustrates the relationship between them.

## RESULTS

**Figure 1:**
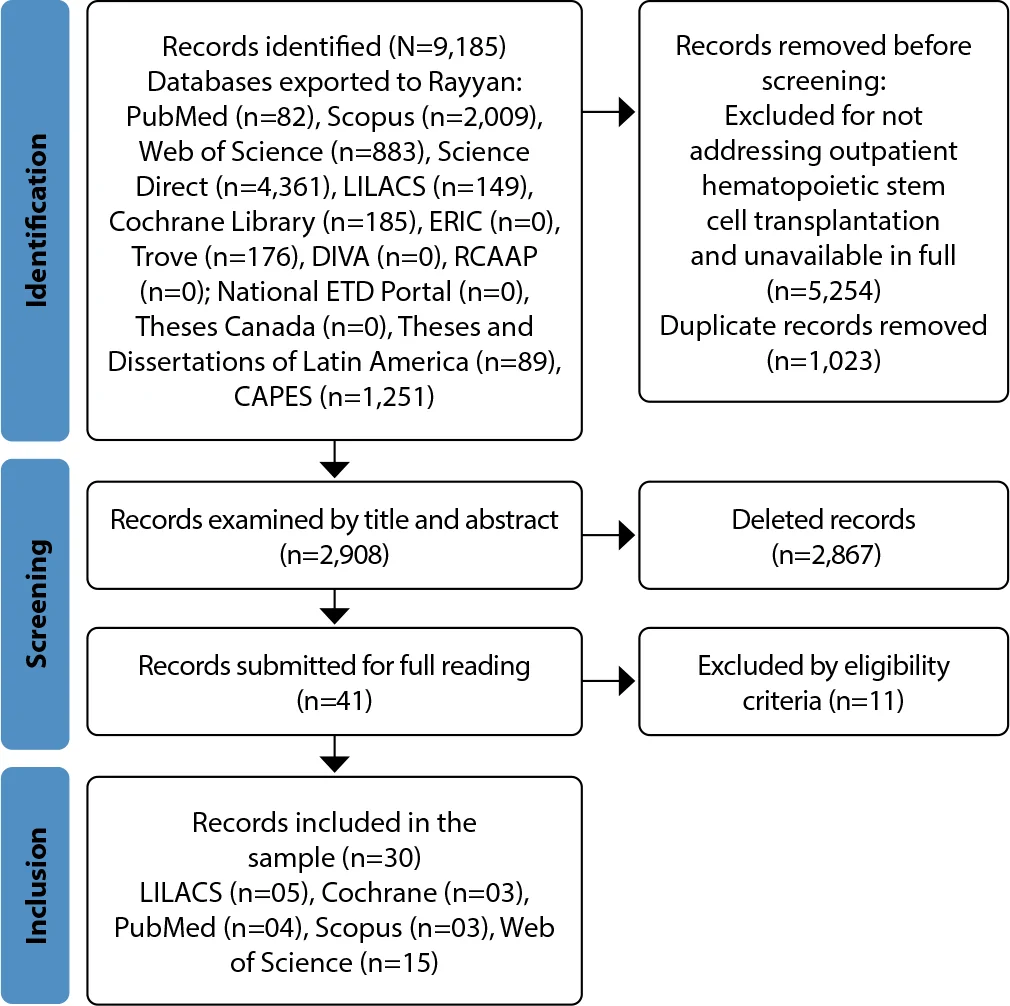
Study selection flowchart, 2025

Originally, the search in data sources and catalogs of theses and dissertations identified 9,185 studies, but 5,254 were not available in full. After that, 2,908 underwent screening of titles and abstracts. Of these, only 41 were submitted to a thorough full-text reading, but, at the end of this process, the study sample consisted of 30 studies. Study selection is presented in the flowchart below (Figure 1).

**Figure 2:**
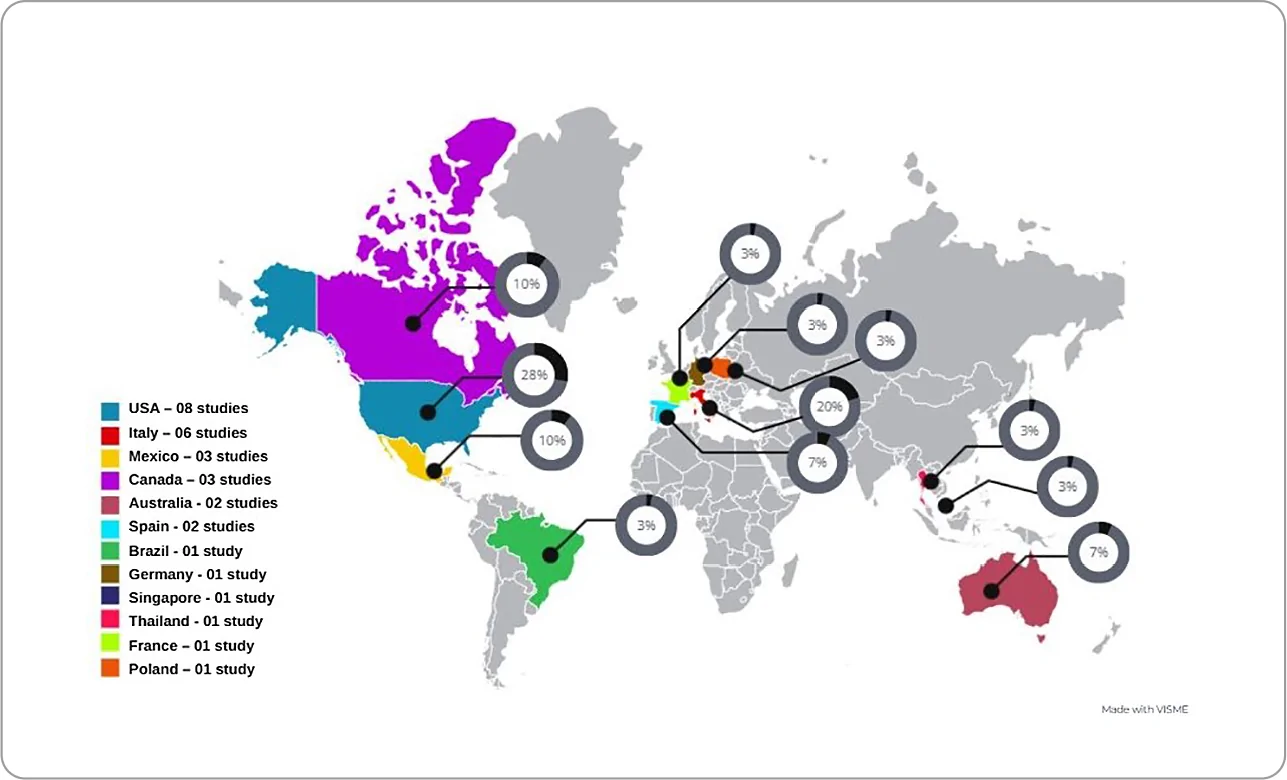
Countries of publications that addressed outpatient hematopoietic stem cell transplantation, 2025

The final sample characterization consisted of 30 studies. As for the country of publication, 12 countries that perform outpatient HSCT were identified, with the highest concentration in the USA, published between 1998 and 2024, with 2019 being the most prominent year, as illustrated in Figure 2. Study characterization regarding the outpatient model and the type of HSCT most frequently performed is described in Chart 2.

**Chart 2:** Study characterization regarding the identification of the type of publication, study title, authorship, year of publication, country where the research was conducted, and type of hematopoietic stem cell transplant performed, Natal, Rio Grande do Norte, Brazil, 2024 (N=30)

ID	Authors/year	Country	HSCT performed	Outpatient model	Metacontext	General context	Specific context	Immediate context
^‡^A1	Meisenberg, BR, Ferran K, Hollenbach K, Brehm T, Jollon J, Piro LD, 1998^(13)^	USA	Autologous	Early discharge and outpatient care		They reduce the burdens and costs associated with HSCT.		Safety in administering high-dose chemotherapy.
^‡^A2	Ruiz-Argüelles GJ, Ruiz-Argüelles A, Pérez-Romano B, Marín-López A, Delgado-Lamas JL, 1998^(14)^	Mexico	Autologous	Total outpatient care		Chemotherapy-induced neutropenia and thrombocytopenia can be safely treated on an outpatient basis, provided certain conditions are met.		Safety in administering high-dose chemotherapy.
^‡^A3	Schulmeister L, Quiett K, Mayer K, 2005^(15)^	USA	Autologous	Total outpatient care		Outpatient HSCT has psychosocial and economic advantages when compared to referral to a transplant center for inpatient treatment.		Safety in administering high-dose chemotherapy.
^‡^A4	Atmar JS, Xá HB, Nash V, 2005^(16)^	USA	Autologous	Total outpatient care			Increased survival rate and time to progression, impacting prognosis.	
^‡^A5	Faucher C, Le Corroller Soriano AG, Esterni B, Vey N, Stoppa AM, Chabannon C *et al*., 2012^(17)^	France	Autologous	Early discharge				
^‡^A6	Martino M, Montanari M, Bruno B, Console G, Irrera G, Messina G *et al*., 2012^(18)^	Italy	Autologous	All models	Outpatient care requires the availability of a permanent outpatient service where medications and supportive care can be provided quickly and efficiently by highly trained personnel.			Safety in administering high-dose chemotherapy.
^‡^A7	Holbro A, Ahmad I, Cohen S, Roy J, Lachance S, Chagnon M *et al*., 2013^(19)^	Canada	Autologous	Total outpatient care		Outpatient HSCT is cost-effective.	Shorter hospital stays.	Safety in administering high-dose chemotherapy.
^II^D1	Barban A, 2013^(4)^	Brazil	Autologous	Early discharge		The need for a caregiver to accompany the patient during HSCT is highly cost-effective.	Significantly reduces hospital stay time and antibiotic exposure, without increasing complications and reducing the number of serious infections.	Safety in administering high-dose chemotherapy. Careful selection of eligible patients.
^‡^A8	Martino M, Montanari M, Ferrara F, Ciceri F, Scortechini I, Palmieri S *et al*., 2014^(20)^	Italy	Autologous	Early discharge		They are highly cost-effective, mainly due to the shorter hospital stay and the reduction in medication administration and laboratory costs.		Outpatient transplantation is feasible, offers clinical advantages, and saves financial resources.
^‡^A9	Campbell P, Walker P, Avery S, Patil S, Curtis D, Schwarer A *et al*., 2014^(21)^	Australia	Allogeneic	Total outpatient care			Significantly reduces hospital stay time in allogeneic HSCT.	Safety in administering high-dose chemotherapy.
^‡^A10	Graff TM, Singavi AK, Schmidt W, Eastwood D, Drobyski WR, Horowitz M *et al*., 2015^(22)^	USA	Autologous	Total outpatient care		Confirmed cost savings in the outpatient setting, due to lower pharmacy, hospitalization, and pathology/laboratory costs.	Minimal mortality with no recurrence, excellent hematopoietic recovery, and acceptable toxicity.	The criteria for patient selection were based on expected adherence, proximity to the oncology center for daily visits, 24-hour caregiver support, favorable performance status, and comorbidity profile.
^‡^A11	Paul TM,Liu SV, Chong EA, Luger SM, Porter DL, Schuster SJ *et al*., 2015^(23)^	USA	Autologous	Early discharge and total outpatient care			Low morbidity and mortality.	Safety in administering chemotherapy at discharge.
^‡^A12	Abid MB, Christopher D, Abid MA, Poon ML, Tan LK, Koh LP *et al*., 2017^(24)^	Singapore	Autologous	Total outpatient care		The total cost of treatment was significantly lower in the outpatient group.	In Asia, it can be considered a treatment with low mortality, lower complication rates, relief from bed restrictions, and fewer infections.	
^‡^A13	Shah N, Cornelison AM, Saliba R, Ahmed S, Nieto YL, Bashir Q *et al*., 2017^(25)^	USA	Autologous	Total outpatient care and late admission			Reduction of adverse events.	Safety in administering high-dose chemotherapy, a safe procedure for patients with multiple myeloma.
^‡^A14	Lisenko K, Sauer S, Bruckner T, Egerer G, Goldschmidt H, Hillengass J *et al*., 2017^(26)^	Germany	Autologous	Total outpatient care	Outpatient care is well established in the USA and Canada, while in Germany and Western Europe, an inpatient setting is the current standard.		Low morbidity, infectious complications, and very high patient satisfaction.	Safety in administering high-dose chemotherapy.
^‡^A15	Martino M, Ciavarella S, De Summa S, Russo L, Meliambro N, Imbalzano L *et al*., 2018^(27)^	Italy	Autologous	Total outpatient care			Improves physical and emotional quality of life.	They increase survival rates by ensuring safety in the introduction of new chemotherapeutic agents.
^‡^A16	Owattanapanich W, Suphadirekkul K, Kunacheewa C, Ungprasert P, Prayongratana K, 2018^(28)^	Thailand	Autologous	Early discharge and total outpatient care		They reduce the burdens and costs associated with HSCT.	The rate of febrile neutropenia and septicemia was lower among patients who received outpatient HSCT.	
^‡^A17	Granot N, Storer BE, Cooper JP, Flowers ME, Sandmaier BM, Storb R, 2019^(29)^	USA	Allogeneic	Total outpatient care and late admission			The incidence rates of neutropenic fever and mucositis are lower in outpatients.	Safety in administering high-dose chemotherapy in older patients.
^‡^A18	Koo J, Silverman S, Nuechterlein B, Keating AK, Verneris MR, Foreman NK *et al*., 2019^(30)^	Italy	Autologous	Total outpatient care			Significantly reduces hospital stay and antibiotic exposure, without increasing complications, and reduces the number of serious infections.	Autologous HSCT for appropriately selected children with central nervous system tumors is safe and effective.
^‡^A19	Kodad SG, Sutherland H, Limvorapitak W, Abou Mourad Y, Barnett MJ, Forrest D *et al*., 2019^(31)^	Canada	Autologous	Total outpatient care			A safe and feasible treatment strategy, with low mortality related to HSCT.	Outpatient care models are needed in the current era due to increased patient volume and limited resources.
^‡^A20	Dhir V, Zibdawi L, Paul HK, Espin-Garcia O, Chen CI, Crump M *et al*., 2019^(32)^	Canada	Autologous	Total outpatient care	Outpatient HSCT has become the standard of care in many centers due to limited resources for inpatient care and increasing financial constraints.	Fatigue levels were significantly higher during outpatient treatment.		
^‡^A21	Dytfeld D, Lojiko-Dankowska A, Nowicki A, Matuszak M, Wache A, Gil L *et al*., 2021^(33)^	Poland	Autologous	Early discharge			Greater ease of life, lower risk of infections, better quality of life, emotional well-being, reduced infections, and lower costs.	Safety in administering high-dose chemotherapy.
^‡^A22	Martino M, Paviglianiti A, Memoli M, Martinelli G, Cerchione C, 2020^(34)^	Italy	Autologous	All models			Improved quality of life for patients.	Patient selection, clinical management, functional status, caregiver support, psychological aspects for identifying eligible patients, outpatient models, and safe administration of high-dose chemotherapy.
^‡^A23	González MJ, Urizar E, Urtaran-Laresgoiti M, Nuño-Solinís R, Lázaro-Pérez E, Vázquez L *et al*., 2021^(35)^	Spain	Allogeneic e autologous	All models	Italian consensus guidelines from 2016 to standardize and fill gaps in aspects of outpatient HSCT.	Risks associated with outpatient procedures have been reported in the literature, such as the high frequency of hospital readmissions due to fever and infections related to neutropenia and graft-versus-host disease in allogeneic HSCT.	Shorter hospital stays, reduced risk of nosocomial infections, and greater patient comfort.	Safety of total outpatient HSCT.
^‡^A24	Martino M, Pitino A, Tripepi G, Paviglianiti A, Russo L, Cusumano G *et* al., 2021^(36)^	Italy	Autologous	Total outpatient care		Cost savings.	Patient preference, reduced exposure to hospital microorganisms, better use of hospital resources.	Most centers that offer outpatient HSCT require a caregiver to be available 24 hours a day, seven days a week, at least during the aplastic phase.
^‡^A25	Gómez-Almaguer D, Gómez-De León A, Colunga-Pedraza PR, Cantú-Rodríguez OG, Gutierrez-Aguirre CH, Ruíz-Arguelles G, 2022^(2)^	Mexico	Allogeneic	Total outpatient care	The cost of hospital-based HSCT is generally very high; therefore, this procedure is more difficult to perform in lowand middle-income countries, such as Mexico. However, performing it outside of a hospital setting is more accessible, safer, and yields similar results to those in high-income countries.	Lower medication costs have been reported in the outpatient setting, with the disadvantage that the patient must live near the clinic.	Infections may be less frequent and severe than with hospital admission, leading to improved quality of life.	Careful patient selection, safety in the administration of oral medications.
^‡^A26	Tan XN, Yew CY, Ragg SJ, Harrup RA, Johnston AM, 2022^(37)^	Australia	Autologous	Total outpatient care and late admission		Outpatient HSCT significantly reduced hospital stay and overall cost. There was no difference in days to neutrophil and platelet engraftment between the inpatient/outpatient groups.		Safety in administering high-dose chemotherapy.
^‡^A27	Jaime-Pérez JC, Meléndez-Flores JD, Ramos-Dávila EM, Hernandez-Coronado M, González-Treviño M, Tarín-Arzaga L *et al*., 2022^(38)^	Mexico	Allogeneic	Total outpatient care	The use of outpatient HSCT in older patients has grown considerably in recent years in developed countries.			The advent of reduced-intensity conditioning has allowed for increased use of outpatient HSCT.
^‡^A28	Marini J, Maldonado A, Weeda E, Neppalli A, Hashmi H, Edwards K, 2023^(39)^	USA	Autologous	Early discharge and total outpatient care		It is safe and reduces the total length of hospital stay and therefore the overall costs of the transplant.	The time to neutrophil and platelet transplantation was shorter in the outpatient group than in the inpatient group.	Safety in administering high-dose chemotherapy.
^‡^A29	Garcés-Carrasco AM, Santacatalina-Roig E, Carretero-Márquez C, Chover-Sierra, Martínez-Sabater A, Balaguer-López E, 2024^(40)^	Spain	Autologous	Home care			It may reduce psychological distress, improve quality of life, and lead to a lower incidence of infections and neutropenic fever.	Safety in administering high-dose chemotherapy.

ID - identification; HSCT - hematopoietic stem cell transplantation;

‡ A - article; USA - United States of America;

II D - dissertation.

**Figure 3:**
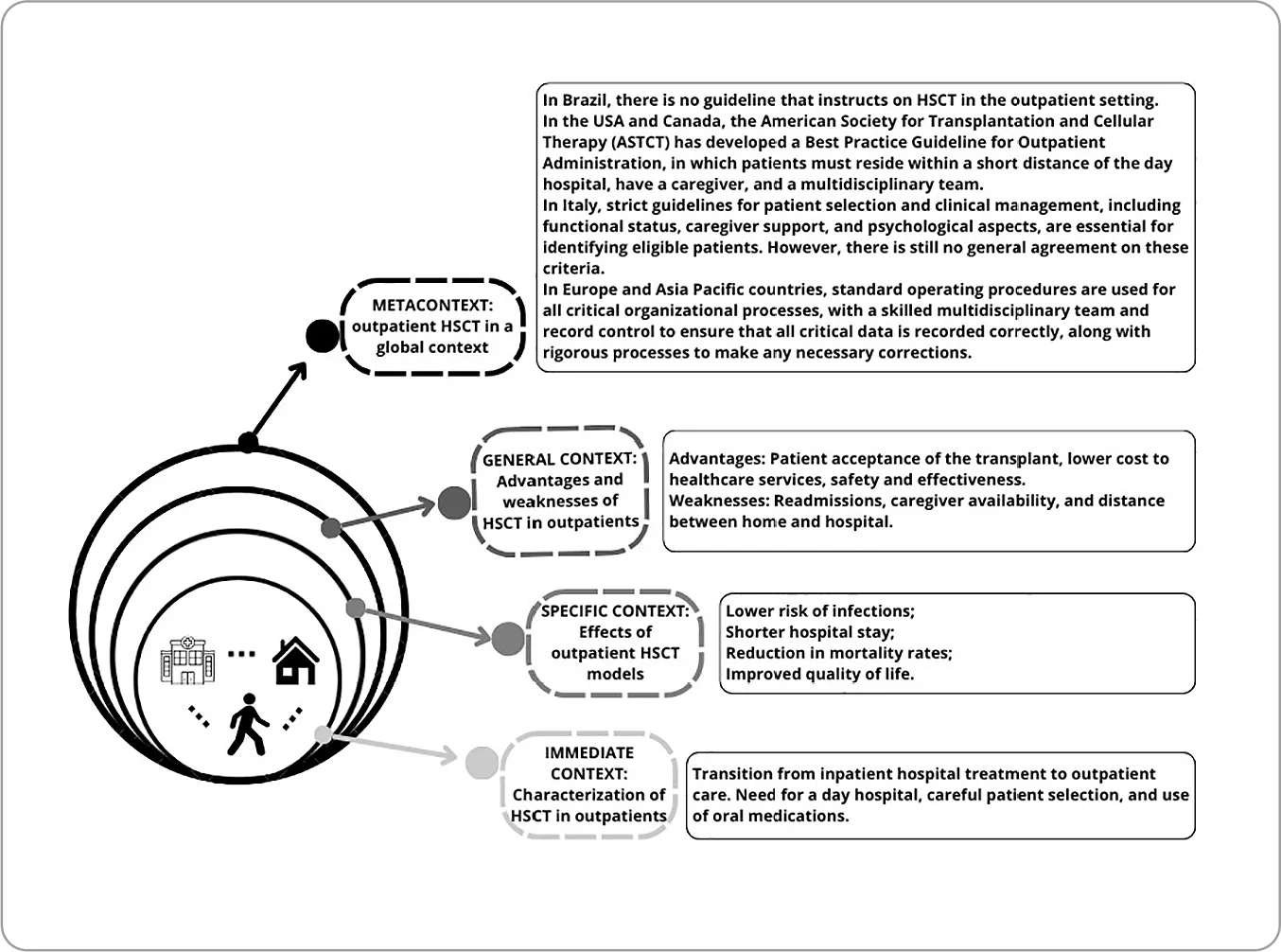
Contextual layers of hematopoietic stem cell transplantation in outpatients, 2025

With regard to contextual layers, Figure 3 presents the main aspects related to metacontext, general context, specific context, and immediate context.

In metacontext, outpatient HSCT was explored in the global context, along with guidelines regulating its implementation in Asian, European, and North American countries. It was observed that each country has its own policies and diverse healthcare organizations.

In the overall context, the advantages and weaknesses of these models for healthcare services were highlighted. Among the advantages, a decrease in the total length of hospital stay, patients’ early return to their family environment, and reduced exposure to antibiotics and other cytotoxic drugs were cited. Among the weaknesses, hospital readmissions and screening based on social criteria for patient admission to outpatient care were mentioned.

In this specific context, the positive effects of these outpatient HSCT models were mentioned, such as reduced costs, lower infection rates, reduction in neutropenic fever, mucositis and positive blood culture results, decreased morbidity and mortality, improved patient physical and emotional well-being, excellent hematopoietic recovery, and accelerated neutrophil and platelet graft engraftment.

In immediate context, the main characteristics were addressed, such as the transition of oncohematological treatment from hospital inpatient care to outpatient care, existing models, and safe practices, such as the administration of high-dose chemotherapy and antimicrobial prophylaxis in HSCT.

## DISCUSSION

HSCT was first performed in 1957 in the USA^(41)^, but its development on an outpatient basis only actually occurred in 1993 among North American countries^(2)^. Such evidence supports the findings of this study.

The results from the contextual analysis proposed by Hinds, Chaves and Cypress^(9)^ are arranged in subtopics organized in each corresponding layer, in order to facilitate understanding of the context in which outpatient HSCT is found.

### Metacontext - outpatient hematopoietic stem cell transplantation in a global context

A significant milestone in oncohematology was the HSCT care transition, which was previously performed in a hospital setting, but can now be safely and economically viable performed on an outpatient basis, adaptable to the realities of various countries around the world^(40)^.

In the meantime, HSCT regulation at the outpatient level is of paramount importance for countries to manage their healthcare services in terms of logistical and financial resources so that they can offer qualified, universal, and safe care that provides good clinical outcomes for patients undergoing this therapy^(42)^.

The use of outpatient HSCT has increased exponentially in recent years in high-income countries compared to middleand low-income countries, and there has also been a greater number of guidelines to guide and regulate this procedure^(38)^. Given this, these countries can foster a model and provide guidelines to encourage the development of HSCT in an outpatient setting in other countries, especially those with limited human and financial resources so that they can create similar standards, but adapted to their particularities and realities^(42)^.

Clinical practice guidelines for outpatient HSCT in Canada and the USA are quite similar regarding patient eligibility and the definition of patient selection criteria^(43)^. In the USA, the American Society for Transplantation and Cellular Therapy has developed a best practice guideline for outpatient management that provides guidance on the responsibilities of the professionals involved, patients, and caregivers, as well as offering recommendations based on scientific evidence^(44)^.

Considering the effectiveness of treatments and the advantages for healthcare services, in Europe, although there is still a predominance of inpatient HSCT, there is already a shift towards home-based complex treatments, such as autologous HSCT. Therefore, Italian consensus guidelines published in 2016 establish rigorous guidelines for screening eligible patients for the outpatient model and clinical management, which include assessment of functional and psychological status and caregiver support^(35)^.

Still within the European context, for the performance of allogeneic HSCT in outpatient centers, accreditation by the European Society for Blood and Marrow Transplantation is necessary as a way to establish professional responsibility towards patients and public healthcare services^(45)^.

HSCT has grown considerably in Asia, according to reports published by the Asia-Pacific Blood and Marrow Transplant Group. Unlike other regions, these countries have seen a higher number of allogeneic transplants compared to autologous transplants in recent years, particularly in countries with larger populations and higher incomes, such as Japan, South Korea, Singapore, and China, compared to those with lower-middle incomes, such as India, Pakistan, and Sri Lanka^(46)^.

In Brazil, no guidelines, laws, or regulations were found in the literature that provide instructions regarding HSCT in an outpatient setting. However, Ordinance 44 of January 2001, from the Ministry of Health, establishes the standards for day-hospital or outpatient care for healthcare services that offer intermediate-level assistance with a maximum patient stay of 12 hours, in addition to providing technical, logistical, and human resources support^(47)^.

It is worth highlighting that, in the Brazilian context, the nursing team plays a fundamental role in outpatient HSCT, as their presence in outpatient clinics and their role in the direct care of oncohematological patients are supported by Resolution 629 of March 2020 from the Federal Nursing Council, which approves and assigns to nurses the provision of more technically complex care that requires specific scientific knowledge for decision-making^(48)^.

### General context - advantages and weaknesses of hematopoietic stem cell transplantation in outpatients

Outpatient HSCT can be considered a less costly treatment, as hospitalization is only indicated if complications need to be treated for both autologous and allogeneic cases. Reduced hospitalization time is beneficial, as it provides a better quality of life for patients, promoting an early return to their family environment^(26,35)^.

Furthermore, the safe administration of high-dose chemotherapy, such as cyclophosphamide, thiotepa, carboplatin, and melphalan, on an outpatient basis stands out, eliminating the need for patient isolation and demonstrating scientifically proven efficacy and safety. This was considered one of the main factors driving the development of outpatient HSCT, and its development has since been encouraged and replicated among HSCT centers^(26)^.

It is worth noting that these chemotherapeutic agents are frequently used in the conditioning of outpatient HSCT, a care procedure performed by nurses in the pre-HSCT period, especially in autologous cases^(5)^. It should be noted that, previously, this stage was performed in a hospital setting, as drugs were administered to promote bone marrow aplasia in order to subsequently infuse HSCs. However, today it is considered a safe procedure in an outpatient setting^(49)^.

Administering high doses of chemotherapy is a task assigned to nursing professionals, as it is considered a technically complex procedure requiring specific knowledge of each medication to be administered, double-checking of medical prescription, patient data, drug information, and dosage. Furthermore, nurses administer the medication and must be able to intervene in possible adverse reactions, as well as provide comfort and guidance regarding the stage of treatment patients are undergoing^(50)^.

Given this milestone, logistical arrangements are essential to ensure adequate care during outpatient treatment. In addition to having a qualified service and a skilled multidisciplinary team, it is crucial that caregivers are available to provide the necessary care, and should be instructed on their responsibilities and how to detect signs of complications in a timely manner^(5,51)^.

Another advantage presented in the results of this study was the lower occurrence of graft-versus-host disease (GVHD) in outpatient allogeneic transplants. This complication leads to impaired functional status, decreased health-related quality of life, financial impacts due to job loss, and the continuous need for immunosuppressive therapy^(52)^.

GVHD commonly occurs in 30 to 60% of patients undergoing allogeneic HSCT, and is associated with a higher number of readmissions, hospitalizations, and outpatient visits, especially among those undergoing multiple lines of chemotherapy and immunosuppressants, such as corticosteroids^(53)^. However, with prophylaxis for GVHD, periodic outpatient follow-up, and reduced exposure to multidrug-resistant microorganisms, a decrease in the occurrence rate of this phenomenon has been observed in patients treated under this new care modality^(51)^.

During nursing care in outpatient HSCT, nurses can utilize educational technologies, such as guides or booklets, to empower patients and family members/caregivers regarding HSCT prevention and the early identification of signs and symptoms of this complication. In addition to providing guidance in a playful and understandable way, these resources are also essential for improving service and optimizing the nursing team’s workflow^(54)^.

Although there are advantages to this therapy, such as reduced hospitalizations and mortality rates, there are also weaknesses, such as hospital readmission. The causes for this phenomenon are related to hemodynamic instability, fever, altered level of consciousness, nausea/vomiting, or unavailability of a caregiver^(55)^.

### Specific context - effects of outpatient hematopoietic stem cell transplantation models

The outpatient HSCT model provides a better quality of life, fewer hospital stays, reduced nosocomial infections, and lower financial costs for healthcare services. Therefore, studies encourage this type of transplant and the dissemination of results demonstrating its effectiveness worldwide^(33)^.

In addition to presenting financial and economic benefits for patients and institutions, it has been observed that patients undergoing HSCT performed entirely during hospitalization experience dissatisfaction with the care provided and a reduction in their quality of life, as the required social isolation leads to high levels of stress^(56)^. However, patients undergoing outpatient HSCT had a significantly lower risk of developing febrile neutropenia and septicemia, greater satisfaction with the care provided, and better quality of life than those admitted for inpatient HSCT^(2,39)^.

However, with healthcare professionals’ improvement, enhancements in chemotherapy infusion devices, verification of the effectiveness of chemotherapy protocols, support in the use of medications, and advances in research, these factors have propelled the transition of onco-hematological treatment from inpatient hospital settings to outpatient settings^(56)^.

From this perspective, it is noteworthy that outpatient transplantation can be considered a feasible option due to its low morbidity and mortality rates^(35,57)^. Furthermore, it was also observed that the time for neutrophil and platelet transplantation is shorter in the outpatient group than in the hospital group^(34)^.

The engraftment period corresponds to a neutrophil count above 500/µL for three consecutive days and a platelet recovery greater than 20,000/µL, without blood transfusion for at least seven days. It is a critical period that requires continuous monitoring to prevent the occurrence of febrile neutropenia^(58)^.

Neutropenia is considered a serious complication that can result in sepsis and even death if not detected and treated promptly. Therefore, nurses should train patients and caregivers to identify signs and symptoms related to this condition for early treatment^(17)^.

Therefore, outpatient HSCT can lead to increased caregiver burden, as this person assumes the responsibility of continuing some of the care provided in healthcare institutions in home environments, periodic follow-up appointments, administration of oral medications, measurement and monitoring of vital signs, and identification of complications^(51)^.

### Immediate context - characterization of hematopoietic stem cell transplantation in outpatients

The most commonly performed transplant in the total outpatient setting is the autologous transplant. In this type of transplant, patientss own HSCs are used, and it is indicated for various oncohematological diseases, such as multiple myeloma, Hodgkin’s lymphoma, non-Hodgkin’s lymphoma, multiple and systemic sclerosis, and some solid tumors^(41)^. It was the first type of HSCT to be performed on an outpatient basis in 1993 in North American countries^(2)^.

In this new care approach, patients undergo all phases of treatment (pre-, Day Zero, and post-) or some of them in an outpatient setting^(5,33)^. In autologous cases, procedures such as HSC mobilization and collection, chemotherapy conditioning, HSC infusion, and post-HSCT care can be performed safely and entirely on an outpatient basis, without causing harm to individuals. These procedures result in greater engraftment success, improved survival, and increased life expectancy^(5)^.

However, for these new services to succeed, a qualified and well-coordinated multidisciplinary team is necessary, composed of hematologists, general practitioners, physiotherapists, pharmacists, psychologists, social workers, and nurses^(57)^. However, the nursing team’s performance stands out, as they follow patients up throughout their entire treatment.

In this context, the nursing team develops specific skills and knowledge that must be constantly updated through continuing health education, involving various teaching activities and knowledge expansion. It is important to highlight that it is essential to articulate the new knowledge acquired with the general strategies applied, in order to promote organizational change focused on the nursing team and the groups of collaborators in the HSCT unit^(59)^.

Healthcare services offering this assistance should operate five to seven days a week for 12 hours a day. They must be linked to the hematology network and have access to laboratories, blood banks, emergency rooms, and inpatient units. In terms of physical structure, they need to include consulting rooms, a procedure room, an infusion room, and a multidisciplinary team specialized in the area^(5,35)^.

Regarding the selection criteria for patients eligible for outpatient HSCT, they must be under 65 years of age, have good vital functions, live near the clinic or in support homes less than 60 minutes away, have regular blood tests, be free of infections, and have a caregiver throughout the treatment^(28,34)^.

It is worth highlighting that the increasing number of outpatient HSCTs has expanded the capacity to provide care for people who need this type of treatment, in a more accessible and low-cost way, but with qualified and safe care^(35)^.

### Study limitations

This study presented limitations regarding the unavailability of fully published studies freely available, which may result in the loss of relevant data. Furthermore, concerning the levels of evidence of the studies, especially those demonstrating the effectiveness of outpatient HSCT in preventing infections and assessing the improvement in survival and quality of life of patients undergoing this type of care, there is a lack of information. Moreover, no studies were found that presented regulations and guidelines for outpatient HSCT implementation.

### Contributions to nursing, health, or public policy

Contributions to the health field included disseminating knowledge about the modalities in which outpatient HSCT occurs and its impacts on patients’ quality of life and on improving the care offered by healthcare services, aiming to consolidate these outpatient models in a more systematic and comprehensive manner.

With regard to nursing, these contributions are geared towards nurses’ practice, since this type of care demands a fundamental role from these professionals for the success of treatment, the execution of complex care, and the provision of more complex care. Therefore, it represents a new field of practice to be explored, capable of demonstrating the importance and value of nursing in this environment.

Therefore, it is necessary to develop new studies that promote discussion about current legislation and guidelines on this topic as a way to encourage the implementation of outpatient HSCT in lowand middle-income countries.

## Data Availability

The research data are available in a repository: https://doi.org/10.17605/OSF.IO/8GSZN.
