# Supplementary material for: Contextual analysis of hematopoietic stem cell transplantation in outpatients
Source: Rev Bras Enferm. 2026 Aug 21;79:e20250173. doi: 10.1590/0034-7167-2025-0173 (PMC13505568; doi:10.1590/0034-7167-2025-0173)
Supplement: Translation

# Translation: Análise contextual do transplante de células-tronco hematopoéticas em pacientes ambulatoriais

**DOI:** 10.1590/0034-7167-2025-0173pt

**Authors:** Camila Caroline Marcolino Soares, Valéria Dantas de Azevedo, Luana Silva Pereira Sátiro, Edmundo João Mansilla-Cordeiro, Viviane Euzébia Pereira Santos, Isabelle Campos de Azevedo

**Affiliations:** I Universidade Federal do Rio Grande do Norte. Natal, Rio Grande do Norte, Brasil

**Keywords:** Pacientes Ambulatoriais, Assistência Ambulatorial, Transplante, Transplante de Células-Tronco Hematopoéticas, Transplante de Medula Óssea.

## Abstract

**Objetivos:**

analisar e mapear na literatura os aspectos contextuais do transplante de células-tronco hematopoéticas em pacientes ambulatoriais.

**Métodos:**

*scoping review* realizada no período de outubro de 2023 a junho de 2024 em bases de dados, portais e biblioteca de teses e dissertações. A coleta e análise de dados ocorreram a partir do referencial de Hinds, Chaves e Cypress, segundo as camadas contextuais metacontexto, geral, específico e imediato.

**Resultados:**

a amostra final foi composta por 30 estudos. Quanto às camadas contextuais, no metacontexto, foi abordado o transplante ambulatorial de células-tronco hematopoéticas no âmbito mundial; no geral, as vantagens e fragilidades; no específico, os efeitos desses modelos assistenciais; e no imediato, a caracterização dos pacientes submetidos ao transplante ambulatorial.

**Considerações Finais:**

observou-se que transplante ambulatorial é uma tendência assistencial que apresenta benefícios para melhoria da saúde dos pacientes, mas que enfrenta desafios para sua implementação.

## INTRODUÇÃO

O transplante de células-tronco hematopoéticas (TCTH) é uma terapêutica indicada para diversos tipos de doenças oncohematológicas, consistindo na infusão de células-tronco hematopoéticas (CTHs) para reestabelecer a função medular para que esta produza novas células saudáveis^(1)^.

Tradicionalmente, esse tratamento costumava ser realizado totalmente em internação hospitalar, pois havia estrutura para assistir esses pacientes e equipamentos necessários para o cuidado^(2)^. Mas com o avanço nas pesquisas científicas na área, o desenvolvimento do TCTH a nível ambulatorial tem sido considerado uma nova opção de terapêutica assistencial segura e viável, se comparada com o transplante realizado integralmente em internação hospitalar^(2,3)^.

No TCTH ambulatorial, o paciente não precisa ficar internado em unidade hospitalar, retornando diariamente ao ambulatório, onde recebe cuidados em todas as fases que envolvem o tratamento, nomeadamente pré-TCTH, Dia Zero e pós-TCTH^(4,5)^. Dessa forma, vivencia uma rotina diária de terapia quimioterápica, transfusional, cuidados com o cateter venoso central e avaliação multidisciplinar até a enxertia medular e alta^(5)^.

Esse modelo exige que o paciente e o cuidador estejam capacitados a identificar sinais de alerta, como febre, sangramentos ou alterações no estado geral, de modo a permitir intervenções precoces e seguras, garantindo a continuidade do tratamento até a enxertia, que ocorre entre dez e 30 dias após a infusão das CTHs. Após a confirmação da enxertia e estabilização clínica, o paciente segue em acompanhamento ambulatorial periódico para controle das complicações a longo prazo e do sucesso do transplante^(6)^.

Destaca-se ainda que, para o êxito do TCTH ambulatorial, é necessário haver atuação conjunta, harmônica e coesa de uma equipe multidisciplinar que trabalha diariamente com orientações sobre nutrição, higiene e administração de medicamentos em domicílio, além de ofertar suporte psicológico e farmacêutico, reforçando a importância da adesão ao tratamento e ao isolamento protetor domiciliar^(3,6)^.

Como membro dessa equipe, os enfermeiros estão presentes ao decorrer de todo o tratamento do paciente. Assim, esses profissionais devem possuir conhecimentos científicos que os habilitem a aplicar os métodos de cuidado de maneira adequada, evidenciando domínio sobre as ações e necessidades do paciente transplantado, o que contribuirá para sua recuperação^(6)^.

Os enfermeiros possuem a responsabilidade de intensificar os cuidados, manter a atenção constante aos riscos, entre outros agravos, adotar estratégias que promovam o autocuidado por parte do paciente, orientar os familiares acerca das necessidades envolvidas nesse processo, e garantir uma comunicação efetiva com a equipe interdisciplinar e multiprofissional^(7)^.

No entanto, mesmo com os benefícios para as instituições de saúde e para o paciente, esse modelo assistencial ainda não é uma realidade praticada pelos serviços de saúde no Brasil e no mundo, pois apresenta uma pequena taxa de adesão, como os Estados Unidos da América (EUA), que apresentam 45% de ambulatórios de TCTH, e o Reino Unido, em que apenas 19% das unidades de tratamento oncohematológico são clínicas ambulatoriais^(8)^.

Diante do exposto, justifica-se a concepção deste estudo, pois o TCTH em âmbito ambulatorial é uma prática assistencial relativamente nova, que necessita ser mais bem elucidada: em que contextos ela ocorre, quais são os motivos que favorecem seu desenvolvimento e quais são os desafios para sua implementação. Tais fatores são cruciais para a interpretação geral do fenômeno investigado, para que ele possa ser reproduzido por outros países.

Para isso, a análise de contexto é um referencial teórico-metodológico que favorece essa investigação, visto que colabora para que os profissionais de saúde compreendam os seres humanos, auxiliando no desenvolvimento de condições para a promoção da saúde e de experiências significativas. Com uma relação proposital, sistemática e analítica com o fenômeno, busca-se o seu significado em sua totalidade e uma análise global. Portanto, a elucidação de um contexto possibilitará a predição, explanação e compreensão do fenômeno investigado^(9)^.

Desse modo, o contexto pode ser definido como um conjunto de relações interconectadas relacionadas a um evento ou cenário. Para realizar sua análise, é necessária a compreensão desse fenômeno em quatro níveis interativos relacionados entre si: metacontexto, contexto geral, específico e imediato^(9)^.

Nessa perspectiva, é importante realizar pesquisa que identifique e analise os aspectos contextuais desse tipo de tratamento ambulatorial para apresentar suas principais características, benefícios, desafios e elucidar a forma de como são realizados mundialmente.

Portanto, delimitou-se a seguinte questão norteadora: quais os aspectos contextuais do TCTH em pacientes ambulatoriais?

## OBJETIVOS

Analisar e mapear na literatura os aspectos contextuais do TCTH em pacientes ambulatoriais.

## MÉTODOS

### Aspectos éticos

A aprovação do Comitê de Ética em Pesquisa foi dispensada, por se tratar de revisão de dados disponíveis na literatura e que não envolveu seres humanos. Mas ressalta-se que todas as citações dos estudos mapeados foram devidamente realizadas e respeitadas.

### Desenho do estudo

Foi desenvolvida *scoping review*, desenvolvida conforme as orientações do *Preferred Reporting Items for Systematic reviews and Meta-Analyses extension for Scoping Reviews* ^(10)^ e as recomendações do JBI^(11)^.

Este tipo de revisão tem como intuito identificar, mapear e sintetizar as evidências e lacunas no conhecimento em torno de um determinado fenômeno. O processo de condução segue o rigor metodológico delineado pelas seguintes etapas: definição do objetivo e da questão de pesquisa; alinhamento dos critérios de inclusão e exclusão com o objetivo do estudo e questão de pesquisa; descrição da busca de evidências, seleção, extração de dados e apresentação dos achados; busca pelas evidências; e seleção, extração, análise, síntese e apresentação das evidências^(11)^.

Vale ressaltar que o método proposto pelo JBI^(11)^ foi aplicado para a elaboração do protocolo do estudo, definição do objetivo, questão de pesquisa, critérios de elegibilidade, busca nas diferentes fontes de dados e mapeamento das evidências alinhadas ao objeto de estudo de maneira organizada e sistematizada. O protocolo foi elaborado e devidamente registrado no *Open Science Framework* (OSF) (DOI: https://doi.org/10.17605/OSF.IO/8GSZN).

### Período e local do estudo

A busca nas bases de dados e na literatura cinzenta foi realizada no período de outubro de 2023 e atualizada em junho de 2024. A coleta ocorreu em bases de dados (*U.S. National Library of Medicine* (PubMed), Scopus, *Web of Science, Science Direct*, Literatura Latino-Americana e do Caribe em Ciências da Saúde (LILACS), Cochrane), em catálogos de teses e dissertações (*Academic Archive Online* (DIVA), *Education Resources Information Center* (ERIC), *DART-Europe E-Theses Portal, Electronic Theses Online Service, National ETD Portal*, Teses e Dissertações da América Latina, Portal de Teses e Dissertações da Coordenação de Aperfeiçoamento de Pessoal de Nível Superior (CAPES)), e em biblioteca digital (*National Library of Australia’s* (Trove)).

### Critérios de inclusão e exclusão

Os critérios de elegibilidade foram definidos a partir da *expertise* e experiência dos membros e pesquisadores do grupo de pesquisa, que, por meio de consenso, decidiram incluir artigos científicos indexados em periódicos da área da saúde, teses e dissertações, pois as evidências são produzidas a partir de métodos científicos robustos, bem delineados com profundidade teórica e que possuem contribuição substancial inovador para o campo científico em estudo.

Esses estudos deveriam responder ao objetivo do estudo e à questão norteadora, estar disponíveis na íntegra em meio eletrônico, sem restrição temporal e de idioma, pelo acesso através da Comunidade Acadêmica Federada via Portal de Periódicos da CAPES do Ministério da Educação. Foram excluídos estudos em formato de editorial, carta ao editor, livro, capítulo de livro, resumo e artigo de opinião. Os documentos duplicados foram considerados apenas uma vez.

### Protocolo do estudo

Antes de iniciar o processo de construção do protocolo, foi realizada uma busca aberta na literatura e no OSF para verificar a existência de estudos já publicados ou se já havia algum protocolo de estudo semelhante em desenvolvimento, o que não foi encontrado.

A elaboração de perguntas de pesquisa que buscam investigar ou avaliar um problema clínico tem como objetivo entender a complexidade do cuidado e difundir esse conhecimento entre a comunidade científica e aos pacientes^(9)^. Portanto, para a elaboração da questão norteadora, foi utilizada a estratégia PCC (População: pacientes; Conceito: TCTH; Contexto: ambulatório). Assim, a questão norteadora determinada para a busca das evidências foi: quais os aspectos contextuais do TCTH em pacientes ambulatoriais?

Sequencialmente, foi feita uma busca por estudos na PubMed e Cochrane com o intuito de verificar os descritores e palavras-chaves que mais se adequavam ao objeto de estudo. Foram selecionados os descritores, em inglês, pelo *Medical Subject Headings*, e em português, por meio do Descritores em Ciências da Saúde. Além disso, utilizaram-se os operadores booleanos *AND* e *OR*. Assim, foi definida a seguinte estratégia de busca em inglês, conforme apresentada no Quadro 1, que foi adaptada de acordo com as especificidades de cada fonte de dados.

**Quadro 1:** Estratégia de busca, 2025

	P - Participantes	C - Conceito	C - Contexto
**Extração**	*Outpatient*	*Transplantation*	*Hematopoietic stem cell transplantation*
**Conversão**	*Outpatient*	*Transplantation; grafting*	*Hematopoietic stem cell transplantation; Bone marrow transplantation; stem cell transplantation*
**Construção**	*Outpatient*	*transplantation OR grafting*	*“hematopoietic stem cell transplantation” OR “bone marrow transplantation” OR “stem cell transplantation”*
**Uso nas bases de dados e catálogos de teses e dissertações**			
***U.S. National Library of Medicine***	*((outpatients[Title/Abstract]) AND (transplantation[Title/Abstract] OR (grafting[Title/Abstract]))) AND (“hematopoietic stem cell transplantation”[Title/Abstract] OR (“bone marrow transplantation”[Title/Abstract] OR “stem cell transplantation”[Title/Abstract]))*		
**Cochrane**	*outpatients in Title Abstract Keyword AND transplantation OR (grafting) in Title Abstract Keyword AND “hematopoietic stem cell transplantation” OR (“bone marrow transplantation” OR “stem cell transplantation”) in Title Abstract Keyword*		
**Coordenação de Aperfeiçoamento de Pessoal de Nível Superior**	*outpatients AND transplantation OR (grafting) AND “hematopoietic stem cell transplantation” OR (“bone marrow transplantation” OR “stem cell transplantation”)*		

As buscas foram iniciadas, e os resultados foram exportados para o *software Rayyan* ^®(12)^, que auxiliou na etapa de pré-análise dos títulos e resumos. Em seguida, os estudos selecionados foram lidos na íntegra e submetidos aos critérios de elegibilidade elencados. As etapas foram realizadas por pares de avaliadores com *expertise* na área de TCTH, e em casos de divergências, foi solicitada a opinião de um terceiro avaliador.

### Análise dos dados

Após a seleção dos estudos, os avaliadores utilizaram um protocolo elaborado para extração das seguintes informações: fontes de dados; título; autores; ano de publicação; país de realização do estudo; delineamento metodológico; objetivo; tipo de TCTH; e as camadas contextuais (metacontexto, contexto geral, contexto específico e contexto imediato). Os dados coletados foram organizados em uma planilha no *software Microsoft Excel^®^* 2017.

Para identificação dos contextos e análise das evidências, foi seguido o referencial teórico-metodológico de análise contextual proposto por Hinds, Chaves e Cypress^(9)^, que permite examinar as relações que constituem um fenômeno para tornar mais compreensível sua ocorrência e extensão em quatro camadas contextuais: metacontexto, contexto geral, específico e imediato^(9)^.

O metacontexto incorpora o conhecimento socialmente construído, destacando o presente e ressaltando as condições e os aprendizados como modelo para o futuro, com explanação e influência direta acerca dos comportamentos e eventos daquele determinado fenômeno^(9)^. Logo, nessa camada, buscaram-se informações acerca de diretrizes, políticas ou outros documentos existentes acerca da implementação do TCTH ambulatorial.

O geral engloba interpretações concebidas por meio das interações passadas e presentes do fenômeno ao longo do tempo^(9)^. Então, buscou-se destacar as vantagens e fragilidades desses modelos para os serviços de saúde. O específico engloba o passado imediato e as circunstâncias que influenciam o momento, como o tempo, pessoas, local, entre outros^(9)^. Assim, procurou-se mapear os efeitos positivos desses modelos de TCTH ambulatoriais.

O contexto imediato se concentra no presente e expressa o fenômeno em questão, podendo ser definido pelos aspectos relevantes do fenômeno, como o espaço, ação e protagonistas, que vão delimitar o padrão de comportamento^(9)^. Nessa camada, foram identificadas as principais características relacionadas ao TCTH ambulatorial.

As evidências foram agrupadas nas camadas contextuais correspondentes em conformidade com o método proposto por Hinds, Chaves e Cypress^(9)^, e apresentadas por meio de uma imagem que ilustra a relação entre elas.

## RESULTADOS

Originalmente, a busca nas fontes de dados e em catálogos de teses e dissertações identificou 9.185 estudos, porém 5.254 não estavam disponíveis na íntegra. Após isso, 2.908 passaram pela triagem de títulos e resumos. Destes, apenas 41 foram submetidos à leitura na íntegra de forma criteriosa, mas, ao final desse processo, a amostra do estudo foi composta por 30 estudos. A seleção dos estudos é apresentada no fluxograma a seguir (Figura 1).

**Figura 1:** Fluxograma de seleção dos estudos, 2025

No que concerne à caracterização da amostra final, esta foi composta por 30 estudos. Quanto ao país de publicação, foram identificados 12 países que realizam o TCTH ambulatorial, com maior concentração nos EUA, publicados entre os anos 1998 e 2024, destacando-se o ano de 2019, como ilustrado na Figura 2. A caracterização dos estudos quanto ao modelo ambulatorial e ao tipo de TCTH mais realizado está descrito no Quadro 2.

**Quadro 2:** Caracterização dos estudos quanto à identificação do tipo de publicação, título do estudo, autoria, ano de publicação, país de realização da pesquisa e tipo de transplante de células-tronco hematopoéticas realizado, Natal, Rio Grande do Norte, Brasil, 2024 (N=30)

ID	Autores/ano	País	TCTH realizado	Modelo ambulatorial	Metacontexto	Contexto geral	Contexto específico	Contexto imediato
^‡^A1	Meisenberg, BR, Ferran K, Hollenbach K, Brehm T, Jollon J, Piro LD, 1998^(13)^	EUA	Autólogo	Alta antecipada e ambulatorial		Reduzem os encargos e custos associados ao TCTH.		Segurança na administração de quimioterapia em altas doses.
^‡^A2	Ruiz-Argüelles GJ, Ruiz-Argüelles A, Pérez-Romano B, Marín-López A, Delgado-Lamas JL, 1998^(14)^	México	Autólogo	Ambulatorial total		Neutropenia e a trombocitopenia induzidas por quimioterapia podem ser tratadas com segurança em nível ambulatorial, desde que certas condições sejam atendidas.		Segurança na administração de quimioterapia em altas doses.
^‡^A3	Schulmeister L, Quiett K, Mayer K, 2005^(15)^	EUA	Autólogo	Ambulatorial total		O TCTH ambulatorial tem vantagens psicossociais e econômicas quando comparado ao encaminhamento para um centro de transplante para tratamento hospitalar.		Segurança na administração de quimioterapia em altas doses.
^‡^A4	Atmar JS, Xá HB, Nash V, 2005^(16)^	EUA	Autólogo	Ambulatorial total			Aumento da taxa de sobrevida e tempo para progressão, impactando o prognóstico.	
^‡^A5	Faucher C, Le Corroller Soriano AG, Esterni B, Vey N, Stoppa AM, Chabannon C *et al*., 2012^(17)^	França	Autólogo	Alta hospitalar precoce				
^‡^A6	Martino M, Montanari M, Bruno B, Console G, Irrera G, Messina G *et al*., 2012^(18)^	Itália	Autólogo	Todos os modelos	O atendimento ambulatorial requer a disponibilidade de um serviço ambulatorial permanente, onde medicamentos e cuidados de suporte possam ser fornecidos de forma rápida e eficiente por pessoal altamente treinado.			Segurança na administração de quimioterapia em altas doses.
^‡^A7	Holbro A, Ahmad I, Cohen S, Roy J, Lachance S, Chagnon M *et al*., 2013^(19)^	Canadá	Autólogo	Ambulatorial total		TCTH ambulatorial é custo-efetivo.	Hospitalizações mais curtas.	Segurança na administração de quimioterapia em altas doses.
^II^D1	Barban A, 2013^(4)^	Brasil	Autólogo	Alta precoce		Necessidade de um cuidador para acompanhar o paciente durante o TCTH é altamente custo-efetivo.	Reduz significativamente o tempo de internação hospitalar e a exposição a antibióticos, sem aumento de complicações e redução do número de infecções graves.	Segurança na administração de quimioterapia em altas doses. Seleção criteriosa dos pacientes elegíveis.
^‡^A8	Martino M, Montanari M, Ferrara F, Ciceri F, Scortechini I, Palmieri S *et al*., 2014^(20)^	Itália	Autólogo	Alta precoce		São altamente custo-efetivos, principalmente devido à menor duração da hospitalização e à redução da administração de medicamentos e dos custos laboratoriais.		O transplante ambulatorial é viável, possui vantagem clínica, bem como a economia de recursos financeiros.
^‡^A9	Campbell P, Walker P, Avery S, Patil S, Curtis D, Schwarer A *et al*., 2014^(21)^	Austrália	Alogênico	Ambulatorial total			Reduz significativamente o tempo de internação hospitalar em TCTH alogênicos.	Segurança na administração de quimioterapia em altas doses.
^‡^A10	Graff TM, Singavi AK, Schmidt W, Eastwood D, Drobyski WR, Horowitz M *et al*., 2015^(22)^	EUA	Autólogo	Ambulatorial total		Economias de custos confirmadas no ambiente ambulatorial, devido a menores custos de farmácia, hospitalização e patologia/laboratório.	Mortalidade mínima sem recidiva, excelente recuperação hematopoiética e toxicidade aceitável.	Os critérios para seleção dos pacientes se basearam na adesão esperada, proximidade do centro oncológico para visitas diárias, apoio 24 horas do cuidador, *status* de desempenho favorável e perfil de comorbidades.
^‡^A11	Paul TM,Liu SV, Chong EA, Luger SM, Porter DL, Schuster SJ *et al*., 2015^(23)^	EUA	Autólogo	Alta precoce e ambulatorial total			Baixa morbidade e mortalidade.	Segurança na administração de quimioterapia em altas.
^‡^A12	Abid MB, Christopher D, Abid MA, Poon ML, Tan LK, Koh LP *et al*., 2017^(24)^	Singapura	Autólogo	Ambulatorial total		O custo total do tratamento foi significativamente menor no grupo ambulatorial.	Pode ser considerado na Ásia um tratamento com baixa mortalidade, menores taxas de complicações, alívio de restrições de leitos e menos infecções.	
^‡^A13	Shah N, Cornelison AM, Saliba R, Ahmed S, Nieto YL, Bashir Q *et al*., 2017^(25)^	EUA	Autólogo	Ambulatorial total e admissão tardia			Redução de eventos adversos.	Segurança na administração de quimioterapia em altas doses, procedimento seguro para pacientes com mieloma múltiplo.
^‡^A14	Lisenko K, Sauer S, Bruckner T, Egerer G, Goldschmidt H, Hillengass J *et al*., 2017^(26)^	Alemanha	Autólogo	Ambulatorial total	Regime ambulatorial está bem estabelecido nos EUA e no Canadá, enquanto na Alemanha e na Europa Ocidental, um o ambiente de internação é o padrão atual.		Baixa morbidade, complicações infecciosas e satisfação muito alta do paciente.	Segurança na administração de quimioterapia em altas doses.
^‡^A15	Martino M, Ciavarella S, De Summa S, Russo L, Meliambro N, Imbalzano L *et al*., 2018^(27)^	Itália	Autólogo	Ambulatorial total			Melhora a qualidade de vida física e emocional.	Aumentam a sobrevida a partir da segurança na introdução de novos quimioterápicos.
^‡^A16	Owattanapanich W, Suphadirekkul K, Kunacheewa C, Ungprasert P, Prayongratana K, 2018^(28)^	Tailândia	Autólogo	Alta precoce e ambulatorial total		Reduzem os encargos e custos associados ao TCTH.	Menor a taxa de neutropenia febril e septicemia entre pacientes que receberam um TCTH ambulatorial.	
^‡^A17	Granot N, Storer BE, Cooper JP, Flowers ME, Sandmaier BM, Storb R, 2019^(29)^	EUA	Alogênico	Ambulatorial total e admissão tardia			As taxas de incidência de febre neutropênica e mucosite são menores em pacientes ambulatoriais.	Segurança na administração de quimioterapia em altas doses em pacientes idosos.
^‡^A18	Koo J, Silverman S, Nuechterlein B, Keating AK, Verneris MR, Foreman NK *et al*., 2019^(30)^	Itália	Autólogo	Ambulatorial total			Reduz significativamente o tempo de internação hospitalar e a exposição a antibióticos, sem aumento de complicações, redução do número de infecções graves.	O TCTH autólogo para crianças adequadamente selecionadas com tumores do sistema nervoso central é seguro e eficaz.
^‡^A19	Kodad SG, Sutherland H, Limvorapitak W, Abou Mourad Y, Barnett MJ, Forrest D *et al*., 2019^(31)^	Canadá	Autólogo	Ambulatorial total			É uma estratégia de tratamento segura e viável, com baixa mortalidade relacionada ao TCTH.	Modelos de atendimento ambulatorial são necessários na era atual devido ao aumento do volume de pacientes e aos recursos limitados.
^‡^A20	Dhir V, Zibdawi L, Paul HK, Espin-Garcia O, Chen CI, Crump M *et al*., 2019^(32)^	Canadá	Autólogo	Ambulatorial total	O TCTH ambulatorial se tornou o padrão de atendimento em muitos centros devido aos recursos limitados para internação e às crescentes restrições financeiras.	Níveis de fadiga foram significativamente maiores durante o tratamento ambulatorial.		
^‡^A21	Dytfeld D, Lojiko-Dankowska A, Nowicki A, Matuszak M, Wache A, Gil L *et al*., 2021^(33)^	Polônia	Autólogo	Alta precoce			Maior facilidade de vida, menor risco de infecções, melhor qualidade de vida, bem-estar emocional, redução de infecções e custos mais baixos.	Segurança na administração de quimioterapia em altas doses.
^‡^A22	Martino M, Paviglianiti A, Memoli M, Martinelli G, Cerchione C, 2020^(34)^	Itália	Autólogo	Todos os modelos			Melhor qualidade de vida dos pacientes.	Seleção de pacientes, manejo clínico, estado funcional, apoio do cuidador, aspectos psicológicos para identificação de pacientes elegíveis, modelos ambulatoriais e administração segura de quimioterapia em altas doses.
^‡^A23	González MJ, Urizar E, Urtaran-Laresgoiti M, Nuño-Solinís R, Lázaro-Pérez E, Vázquez L *et al*., 2021^(35)^	Espanha	Alogênico e autólogo	Todos os modelos	Diretrizes de consenso italianas em 2016 para homogeneizar e preencher lacunas nos aspectos do TCTH ambulatorial.	Riscos associados às modalidades ambulatoriais têm sido relatados na literatura, como a alta frequência de readmissões hospitalares por febre e infecções relacionadas à neutropenia e doença do enxerto contra o hospedeiro em TCTH alogênicos.	Menor tempo de internação hospitalar e menos riscos de infecções nosocomiais e maior conforto para os pacientes.	Segurança do TCTH ambulatorial total.
^‡^A24	Martino M, Pitino A, Tripepi G, Paviglianiti A, Russo L, Cusumano G *et* al., 2021^(36)^	Itália	Autólogo	Ambulatorial total		Economia de custos.	Preferência do paciente, exposição reduzida a microrganismos hospitalares, melhor uso dos recursos hospitalares.	A maioria dos centros que oferecem TCTH ambulatorial exige a disponibilidade de um cuidador por 24 horas por dia, sete dias por semana, pelo menos durante a fase aplástica.
^‡^A25	Gómez-Almaguer D, Gómez-De León A, Colunga-Pedraza PR, Cantú-Rodríguez OG, Gutierrez-Aguirre CH, Ruíz-Arguelles G, 2022^(2)^	México	Alogênico	Ambulatorial total	O custo do TCTH hospitalar é geralmente muito alto; portanto, esse procedimento é mais difícil de ser realizado em países de baixa e média renda, como o México. Contudo, a realização fora do ambiente hospitalar é mais acessível, seguro e dotado de resultados semelhantes aos países de alta renda.	Custos mais baixos com medicamentos foram relatados no ambiente ambulatorial, tendo como desvantagem o fato que o paciente deve morar próximo ao ambulatório.	As infecções podem ser menos frequentes e graves do que em internação hospitalar, com a melhora na qualidade de vida.	Seleção cuidadosa dos pacientes, segurança na administração de medicamentos orais.
^‡^A26	Tan XN, Yew CY, Ragg SJ, Harrup RA, Johnston AM, 2022^(37)^	Austrália	Autólogo	Ambulatorial total e admissão tardia		TCTH ambulatoriais levaram a reduziu significativamente os dias de internação e o custo geral. Não houve diferença nos dias até o enxerto de neutrófilos e plaquetas entre os grupos de pacientes internados/ambulatoriais.		Segurança na administração de quimioterapia em altas doses.
^‡^A27	Jaime-Pérez JC, Meléndez-Flores JD, Ramos-Dávila EM, Hernandez-Coronado M, González-Treviño M, Tarín-Arzaga L *et al*., 2022^(38)^	México	Alogênico	Ambulatorial total	A realização de TCTH ambulatorial em idosos cresceu consideravelmente nos últimos anos nos países desenvolvidos.			O advento do condicionamento de intensidade reduzida permitiu o aumento do uso de TCTH ambulatorial.
^‡^A28	Marini J, Maldonado A, Weeda E, Neppalli A, Hashmi H, Edwards K, 2023^(39)^	EUA	Autólogo	Alta precoce e ambulatorial total		É seguro e reduz o tempo total de internação hospitalar e, portanto, os custos gerais do transplante.	O tempo para enxerto de neutrófilos e plaquetas foi menor no grupo ambulatorial do que no grupo hospitalar.	Segurança na administração de quimioterapia em altas doses.
^‡^A29	Garcés-Carrasco AM, Santacatalina-Roig E, Carretero-Márquez C, Chover-Sierra, Martínez-Sabater A, Balaguer-López E, 2024^(40)^	Espanha	Autólogo	Domiciliar			Pode reduzir o sofrimento psicológico, melhorar a qualidade de vida, menor ocorrência de infecções e febre neutropênica.	Segurança na administração de quimioterapia em altas doses.

* ID - identificação;

† TCTH - transplante de células-tronco hematopoéticas;

‡ A - artigo;

§ EUA - Estados Unidos da América;

II D - dissertação.

**Figura 2:** Países das publicações que abordaram o transplante de células-tronco hematopoéticas ambulatorial, 2025

No que se referem às camadas contextuais, a Figura 3 apresenta os principais aspectos relacionados ao metacontexto, contexto geral, específico e imediato.

**Figura 3:** Camadas contextuais do transplante de células-tronco hematopoéticas em pacientes ambulatoriais, 2025

No metacontexto, foram explorados o TCTH ambulatorial no cenário mundial e diretrizes que regulamentam sua realização nos países asiáticos, europeus e norte-americanos. Percebeu-se que cada país possui suas políticas próprias e organizações assistenciais diversas.

No contexto geral, destacaram-se as vantagens e fragilidades desses modelos para os serviços de saúde. Entre as vantagens, foram citados o decréscimo do tempo total de internação hospitalar, o retorno precoce do paciente para seu âmbito familiar, e a redução da exposição a antibiótico e a outros medicamentos citotóxicos. Entre as fragilidades, foram citadas as readmissões hospitalares e triagem dos critérios sociais para admissão do paciente à assistência ambulatorial.

No contexto específico, foram mencionados os efeitos positivos desses modelos de TCTH ambulatoriais, como a redução de custos, menores taxas de infecções, redução da febre neutropênica, mucosite e de resultados de hemoculturas positivas, diminuição da morbidade e mortalidade, melhoria do bem-estar físico e emocional do paciente, excelente recuperação hematopoética, e aceleramento da pega do enxerto de neutrófilos e plaquetas.

No contexto imediato, foram abordadas as principais características, como a transição do tratamento oncohematológico da internação hospitalar para o nível ambulatorial, os modelos existentes e práticas seguras, como a administração da quimioterapia em altas doses e da profilaxia antimicrobiana no TCTH.

## DISCUSSÃO

O TCTH foi realizado a primeira vez em 1957 nos EUA^(41)^, mas seu desenvolvimento em âmbito ambulatorial só ocorreu de fato em 1993 entre países da América do Norte^(2)^. Tais evidências corroboram os achados deste estudo.

Os resultados provenientes da análise contextual proposta por Hinds, Chaves e Cypress^(9)^ estão dispostos nos subtemas organizados em cada camada correspondente, com o intuito de facilitar a compreensão acerca do contexto em que se encontra o TCTH ambulatorial.

### Metacontexto - o transplante de células-tronco hematopoéticas ambulatorial no contexto mundial

Um marco importante na área da oncohematologia foi a transição da assistência do TCTH, que antes era realizado em internação hospitalar, mas agora há a possibilidade de ser desenvolvido a nível ambulatorial de forma segura, sendo viável economicamente e adaptável às realidades de diversos países do mundo^(40)^.

Nesse ínterim, a regulamentação do TCTH a nível ambulatorial é de suma importância para que os países gerenciem seus serviços de saúde quanto aos recursos logísticos e financeiros, para que eles possam ofertar uma assistência qualificada, de maneira universal, segura e que proporcione um bom desempenho clínico do paciente submetido a essa terapêutica^(42)^.

A realização do TCTH ambulatorial aumentou exponencialmente nos últimos anos em países de alta renda, quando comparados àqueles de média e baixa renda, além de verificar o maior número de diretrizes para orientar e regulamentar esse procedimento^(38)^. Diante disso, esses países podem fomentar um modelo e disponibilizar orientações para incentivar o desenvolvimento do TCTH em ambiente ambulatorial em outros países, principalmente daqueles que possuem recursos humanos e financeiros limitados, para que eles criem normas semelhantes, mas adaptadas às suas particularidades e realidades^(42)^.

As diretrizes de prática clínica para TCTH ambulatorial no Canadá e nos EUA são bastante semelhantes quanto à elegibilidade dos pacientes e definição dos critérios de seleção destes^(43)^. Nos EUA, a *American Society for Transplantation and Cellular Therapy* elaborou uma diretriz de melhores práticas para administração ambulatorial que dispõe de orientações acerca das atribuições dos profissionais envolvidos, pacientes e cuidadores, além de ofertar recomendações baseadas em evidências científicas^(44)^.

Ao considerar a eficácia dos tratamentos e as vantagens para os serviços de saúde, na Europa, embora ainda haja uma predominância do TCTH em internação hospitalar, já se é verificada uma transferência de tratamentos complexos para o domicílio, como o TCTH autólogo. Dessa maneira, as diretrizes de consenso italianas publicadas em 2016 estabelecem orientações rigorosas para a triagem de pacientes elegíveis ao modelo ambulatorial e manejo clínico, os quais incluem a avaliação do estado funcional, psicológico e apoio do cuidador^(35)^.

Ainda no contexto europeu, para a realização de TCTHs alogênicos em centros ambulatoriais, faz-se necessária a acreditação pela *European Society for Blood and Marrow Transplantation* como forma de estabelecer uma responsabilidade profissional para com os pacientes e com os serviços de saúde pública^(45)^.

O TCTH tem crescido consideravelmente no continente asiático, conforme informações publicadas pelos relatórios do Grupo de Transplante de Sangue e Medula Óssea da Ásia-Pacífico. Diferentemente de outros cenários, nesses países, é verificado um maior número de transplantes alogênicos em detrimento do tipo autólogo nos últimos anos, principalmente entre países com maior base populacional e com elevado rendimento financeiro, como o Japão, a Coréia do Sul, a Singapura e a China, quando comparados com que aqueles de renda média/baixa, como a Índia, o Paquistão e o Sri Lanka^(46)^.

No Brasil, não foi encontrada na literatura nenhuma diretriz, lei ou regulamentação que instrua acerca do TCTH no contexto ambulatorial. No entanto, a Portaria nº 44, de janeiro de 2001, do Ministério da Saúde, estabelece as normas para o atendimento em regime de hospital-dia ou ambulatorial para aquele serviço de saúde que dispõe de assistência de nível intermediário com o período de permanência do paciente de, no máximo, 12 horas, além de ofertar suporte técnico, logístico e de recursos humanos^(47)^.

Vale destacar que, no contexto brasileiro, a equipe de enfermagem tem fundamental importância no TCTH ambulatorial, pois sua presença nos ambulatórios e sua atuação no cuidado direto aos pacientes oncohematológico são respaldadas pela Resolução nº 629, de março de 2020, do Conselho Federal de Enfermagem, que aprova e atribui ao enfermeiro a prestação de cuidados mais complexos tecnicamente e que exigem conhecimentos científicos específicos para tomada de decisão^(48)^.

### Contexto geral - vantagens e fragilidades do transplante de células-tronco hematopoéticas em pacientes ambulatoriais

O TCTH a nível ambulatorial pode ser considerado um tratamento menos custoso, pois a internação hospitalar só é indicada se houver necessidade de tratamento de complicações, tanto para casos autólogos quanto para alogênicos. A redução do tempo de internação se torna benéfica, visto que proporciona uma melhor qualidade de vida ao paciente, promovendo o retorno ao seu ambiente familiar precoce^(26,35)^.

Além disso, destaca-se a administração segura da quimioterapia em altas doses, como a ciclofosfamida, tiotepa, carboplatina e melfalano, em ambulatório, sem que o paciente precise ficar em isolamento protetor para recebê-la, com eficácia e segurança comprovadas cientificamente. Esse foi considerado um dos principais fatores que impulsionaram a realização do TCTH ambulatorial, e desde então seu desenvolvimento tem sido incentivado e reproduzido entre os centros transplantadores de CTHs^(26)^.

Ressalta-se que esses quimioterápicos são frequentemente utilizados no condicionamento do TCTH ambulatorial, um cuidado executado pelos enfermeiros no período pré-TCTH, principalmente em casos autólogos^(5)^. Salienta-se que, antes, essa etapa era realizada em regime de internação hospitalar, pois são fármacos administrados com o intuito de promover a aplasia medular para posteriormente infundir as CTHs, porém hoje se configura como um procedimento seguro em ambiente ambulatorial^(49)^.

A administração de quimioterápicos em altas doses é um cuidado atribuído ao profissional enfermeiro, pois é considerado um procedimento de maior complexidade técnica, a qual exige conhecimentos específicos de cada medicação a ser administrada, dupla conferência da prescrição médica, dos dados do paciente, do fármaco e da dosagem. Além disso, ainda administra a medicação e deve estar apto para intervir em possíveis reações adversas, além de promover conformou e orientar acerca da etapa do tratamento a qual o paciente está sendo submetido^(50)^.

Diante desse marco, arranjos logísticos são imprescindíveis para que seja realizado o cuidado adequado durante o tratamento ambulatorial. Além de possuir um serviço habilitado e uma equipe multiprofissional qualificada, é de suma importância que o cuidador esteja disponível para realizar os cuidados necessários, devendo ser orientado sobre suas atribuições e sobre como detectar sinais de intercorrências em tempo hábil^(5,51)^.

Outra vantagem apresentada nos resultados deste estudo foi a menor ocorrência da doença do enxerto contra o hospedeiro (DECH) em transplantes alogênicos ambulatoriais. Essa complicação acarreta no comprometimento de estado funcional, diminuição da qualidade de vida relacionada à saúde, impactos financeiros devido à perda de emprego, e necessidade contínua de terapia imunossupressora^(52)^.

A DECH costuma ocorrer em 30 a 60% dos pacientes submetidos ao TCTH alogênico, e está associada ao maior número de readmissões, internações e de consultas ambulatoriais, principalmente entre aqueles submetidos a linhas múltiplas de quimioterápicos e imunossupressores, como os corticosteroides^(53)^. No entanto, com a profilaxia para a DECH, o acompanhamento ambulatorial periódico e a menor exposição a microrganismos multirresistentes verificaram uma redução da taxa de ocorrência desse fenômeno em pacientes atendidos nessa nova modalidade assistencial^(51)^.

Durante os cuidados de enfermagem no TCTH ambulatorial, o enfermeiro pode buscar tecnologias educacionais, como guias ou cartilhas, para capacitar os pacientes e familiares/cuidadores quanto à prevenção da DECH e também à identificação precoce de sinais e sintomas dessa complicação. Além de ofertar orientações de forma lúdica e compreensível, tais recursos também são imprescindíveis para melhorar o serviço e otimizar o processo de trabalho da equipe de enfermagem^(54)^.

Embora existam vantagens acerca dessa terapêutica, como a redução de internações hospitalares e da taxa de mortalidade, são verificadas também fragilidades, como a readmissão hospitalar. As causas para esse fenômeno estão relacionadas à instabilidade hemodinâmica, febre, alteração do nível de consciência, náusea/vômito ou indisponibilidade de um cuidador^(55)^.

### Contexto específico - efeitos dos modelos de transplante de células-tronco hematopoéticas ambulatorial

O modelo de TCTH ambulatorial proporciona uma melhor qualidade de vida, menos episódios de internação hospitalar, redução de infecções nosocomiais e menor custo financeiro para os serviços de saúde. Dessa maneira, os estudos incentivam esse tipo de transplante e a divulgação de resultados que demonstrem a sua eficácia a nível mundial^(33)^.

Além de apresentar benefícios financeiros e econômicos para os pacientes e instituições, é verificado que os pacientes submetidos ao TCTH realizado integralmente em internação hospitalar apresentam insatisfação da assistência prestada e redução da qualidade de vida, pois o isolamento social exigido leva às pessoas a níveis de estresse elevados^(56)^. Contudo, os pacientes submetidos ao TCTH ambulatorial apresentaram um risco significativamente menor para desenvolver neutropenia febril e septicemia, maior satisfação com a assistência prestada e melhor qualidade de vida do que aqueles admitidos para TCTH em internação hospitalar^(2,39)^.

Entretanto, com o aperfeiçoamento dos profissionais de saúde, melhorias nos dispositivos de infusão para quimioterapia, verificação da eficácia dos protocolos quimioterápicos, suporte no uso das medicações e avanços das pesquisas, tais fatores alavancaram a transição do tratamento onco-hematológico em internação hospitalar para o ambiente ambulatorial^(56)^.

Destaca-se, nessa perspectiva, que o transplante ambulatorial pode ser considerado uma opção factível por apresentar baixa morbidade e mortalidade^(35,57)^. Ademais, também é verificado que o tempo para enxerto de neutrófilos e plaquetas é menor no grupo de pacientes ambulatoriais do que no grupo hospitalar^(34)^.

O período de enxertia corresponde à contagem de neutrófilos acima que 500/µL durante três dias consecutivos e recuperação plaquetária maior que 20.000/µL, sem realizar transfusão sanguínea por pelo menos durante sete dias. É um período crítico, que precisa de monitoramento contínuo para evitar a ocorrência de neutropenia febril^(58)^.

A neutropenia é considerada uma complicação grave que pode resultar em sepse e até óbito, se não detectada e tratada em tempo oportuno. Nesse ínterim, o enfermeiro deve capacitar o paciente e cuidador para identificar sinais e sintomas relacionados a essa condição para o tratamento precoce^(17)^.

Diante disso, o TCTH ambulatorial pode apresentar um aumento na sobrecarga do cuidador, uma vez que essa pessoa assume a responsabilidade de continuar alguns cuidados realizados em instituições de saúde no ambiente domiciliar, acompanhamento periódico em consultas, além da administração de medicamentos orais, aferição e monitoramento dos sinais vitais, e identificação de intercorrências^(51)^.

### Contexto imediato - caracterização do transplante de células-tronco hematopoéticas em pacientes ambulatoriais

O transplante mais realizado na modalidade ambulatorial total é o autólogo. Nesse tipo de transplante, são utilizadas as próprias CTHs do paciente, e é indicado para diversas doenças oncohematológicas, como o mieloma múltiplo, linfoma de Hodgkin, linfoma não Hodgkin, esclerose múltipla e sistêmica, e alguns tumores sólidos^(41)^. Ele foi o primeiro tipo de TCTH a ser realizado a nível ambulatorial no ano de 1993 em países da América do Norte^(2)^.

Nessa nova abordagem assistencial, os pacientes realizam todas as fases do tratamento (pré-, Dia Zero e pós-) ou alguma delas em ambiente ambulatorial^(5,33)^. Nos casos autólogos, procedimentos como a mobilização e a coleta das CTHs, o condicionamento quimioterápico, a infusão das CTHs e os cuidados pós-TCTH podem ser realizados de maneira segura e totalmente em ambulatório, sem causar danos ao indivíduo. Esses procedimentos têm como consequência o maior êxito na enxertia, a melhoria da sobrevida e o aumento da expectativa de vida^(5)^.

No entanto, para que esses novos serviços obtenham êxito, é necessário a atuação de uma equipe multiprofissional qualificada e articulada composta por médicos hematologistas, clínicos gerais, fisioterapeutas, farmacêuticos, psicólogos, assistentes sociais e enfermeiros^(57)^. Porém, destaca-se a atuação da equipe de enfermagem, por acompanhar o paciente no decorrer de todo seu tratamento.

Nesse contexto, a equipe de enfermagem desenvolve habilidades e conhecimentos específicos que devem ser constantemente atualizados por meio da educação permanente em saúde, envolvendo diversas atividades de ensino e ampliação do saber. É importante destacar que é essencial articular os novos conhecimentos adquiridos com as estratégias gerais aplicadas, para que se promova uma mudança organizacional voltada à equipe de enfermagem e aos grupos de colaboradores na unidade de TCTH^(59)^.

Os serviços de saúde que oferecem essa assistência devem funcionar entre cinco e sete dias semanais por 12 horas diárias. Devem estar vinculados à rede hematológica e ter acesso a laboratórios, banco de sangue, unidades de pronto-socorro e internação. Quanto à estruturação física, precisam contar com a presença de consultórios, sala de procedimentos e de infusão, além de uma equipe multidisciplinar especializada na área^(5,35)^.

Quanto aos critérios de seleção dos pacientes elegíveis ao TCTH ambulatorial, estes devem ter idade inferior a 65 anos, estar com boa funcionalidade vital, morar próximo ao ambulatório ou em casas de apoio com uma distância inferior a 60 minutos, coletar exames periodicamente, não apresentar infecções, e possuir um cuidador durante todo o tratamento^(28,34)^.

Ressalta-se que o número crescente de TCTH ambulatorial ampliou a capacidade de atendimento para pessoas que necessitam desse tipo de tratamento, de modo mais acessível, com baixo custo, mas com atenção qualificada e segura^(35)^.

### Limitações do estudo

Este estudo apresentou limitações referentes à indisponibilidade de estudos publicados na íntegra de forma gratuita, o que pode resultar na perda de dados relevantes. Além disso, no que diz respeito aos níveis de evidência dos estudos, principalmente daqueles que demonstram a eficácia do TCTH ambulatorial na prevenção de infecções e que avaliam a melhoria da sobrevida e da qualidade de vida dos pacientes submetidos a essa modalidade assistencial, há uma carência de informações. Além disso, não foram encontrados estudos que apresentaram normativas e orientações para a implementação do TCTH ambulatorial.

### Contribuições para a área da enfermagem, saúde ou políticas públicas

As contribuições para a área da saúde incluíram a disseminação de conhecimentos sobre as modalidades em que ocorre o TCTH ambulatorial e seus impactos na qualidade de vida dos pacientes e na melhoria da assistência oferecida pelos serviços de saúde, visando consolidar esses modelos ambulatoriais de maneira mais sistematizada e ampliada.

No que se refere à enfermagem, trata-se de contribuições voltadas para a prática dos enfermeiros, pois essa modalidade assistencial exige um papel fundamental desses profissionais para o êxito do tratamento, a execução de cuidados complexos e a prestação de cuidados de maior complexidade. Portanto, representa um novo campo de prática a ser explorado, capaz de demonstrar a importância e a valorização da enfermagem nesse ambiente.

Portanto, faz-se necessário o desenvolvimento de novos estudos que promovam a discussão acerca das legislações e diretrizes vigentes acerca dessa temática como maneira de fomentar a implantação do TCTH ambulatorial em países de média e baixa renda.

## Data Availability

Os dados de pesquisa estão disponíveis em repositório: https://doi.org/10.17605/OSF.IO/8GSZN.
